# Hybrid Deep Learning (hDL)-Based Brain-Computer Interface (BCI) Systems: A Systematic Review

**DOI:** 10.3390/brainsci11010075

**Published:** 2021-01-08

**Authors:** Nibras Abo Alzahab, Luca Apollonio, Angelo Di Iorio, Muaaz Alshalak, Sabrina Iarlori, Francesco Ferracuti, Andrea Monteriù, Camillo Porcaro

**Affiliations:** 1 Department of Information Engineering, Università Politecnica delle Marche, Via Brecce Bianche 12, 60131 Ancona, Italy; nibras.abo.alzahab@gmail.com (N.A.A.); apolloniol22@gmail.com (L.A.); angelo.di-iorio94@hotmail.it (A.D.I.); Mouazalshalak@gmail.com (M.A.); s.iarlori@staff.univpm.it (S.I.); f.ferracuti@univpm.it (F.F.); a.monteriu@staff.univpm.it (A.M.); 2 Institute of Cognitive Sciences and Technologies (ISTC)—National Research Council (CNR), 00185 Rome, Italy; 3 S. Anna Institute and Research in Advanced Neurorehabilitation (RAN), 88900 Crotone, Italy; 4 Centre for Human Brain Health, School of Psychology, University of Birmingham, Birmingham B15 2TT, UK; 5 Research Center for Motor Control and Neuroplasticity, KU Leuven, 3000 Leuven, Belgium

**Keywords:** Brain-Computer Interface (BCI), Hybrid Deep Learning, Electroencephalography (EEG), Neural Networks, review, survey

## Abstract

Background: Brain-Computer Interface (BCI) is becoming more reliable, thanks to the advantages of Artificial Intelligence (AI). Recently, hybrid Deep Learning (hDL), which combines different DL algorithms, has gained momentum over the past five years. In this work, we proposed a review on hDL-based BCI starting from the seminal studies in 2015. Objectives: We have reviewed 47 papers that apply hDL to the BCI system published between 2015 and 2020 extracting trends and highlighting relevant aspects to the topic. Methods: We have queried four scientific search engines (Google Scholar, PubMed, IEEE Xplore and Elsevier Science Direct) and different data items were extracted from each paper such as the database used, kind of application, online/offline training, tasks used for the BCI, pre-processing methodology adopted, type of normalization used, which kind of features were extracted, type of DL architecture used, number of layers implemented and which optimization approach were used as well. All these items were then investigated one by one to uncover trends. Results: Our investigation reveals that Electroencephalography (EEG) has been the most used technique. Interestingly, despite the lower Signal-to-Noise Ratio (SNR) of the EEG data that makes pre-processing of that data mandatory, we have found that the pre-processing has only been used in 21.28% of the cases by showing that hDL seems to be able to overcome this intrinsic drawback of the EEG data. Temporal-features seem to be the most effective with 93.94% accuracy, while spatial-temporal features are the most used with 33.33% of the cases investigated. The most used architecture has been Convolutional Neural Network-Recurrent Neural Network CNN-RNN with 47% of the cases. Moreover, half of the studies have used a low number of layers to achieve a good compromise between the complexity of the network and computational efficiency. Significance: To give useful information to the scientific community, we make our summary table of hDL-based BCI papers available and invite the community to published work to contribute to it directly. We have indicated a list of open challenges, emphasizing the need to use neuroimaging techniques other than EEG, such as functional Near-Infrared Spectroscopy (fNIRS), deeper investigate the advantages and disadvantages of using pre-processing and the relationship with the accuracy obtained. To implement new combinations of architectures, such as RNN-based and Deep Belief Network DBN-based, it is necessary to better explore the frequency and temporal-frequency features of the data at hand.

## 1. Introduction

The history of Brain-Computer Interfaces (BCIs) developed from the days of early digital technology to today’s highly sophisticated approaches for signal detection, recording, and analysis [1]. In recent years, it has attracted increasing attention from academics and the public due to its potential clinical applications [2]. BCI is a technology that translates signals generated by brain activity into control signals without the involvement of peripheral nerves and muscles and uses these signals to control external devices [3].

The BCI system is composed of different consecutive processes, which are sequenced as signal acquisition, extraction of the desired features from the task, selection of more relevant subset from the feature set, classification of the mental state, and generated feedback signals. These brain signals are extracted, decoded, and studied with the help of various monitoring non-invasive techniques like electroencephalography (EEG), functional magnetic resonance imaging (fMRI), and functional near-infrared spectroscopy (fNIRS) among others [4].

Among those neuroimaging techniques, EEG has several advantages in a BCI environment since it is portable, relatively inexpensive (especially if compared with fMRI), and easy to use with high temporal resolution. The optimal temporal information and the direct measure of the neuronal activity provided by EEG are strongly recommended, especially in BCI involving real-time neurofeedback. In this respect, EEG overcomes the main fMRI and fNIRS low temporal resolution limitation intrinsically related to those techniques that indirectly measure the brain activity based on the principle of neurovascular coupling that measures the increase in regional cerebral blood flow (i.e., increase in oxygenated and decrease in deoxygenated hemoglobin) induced by neuronal activation.

These techniques suffer, in their nature, from a low Signal-to-Noise Ratio (SNR) [5], as brain activity is often affected by multiple sources of environmental, physiological, and activity-specific noise, called ‘artifacts’ [6,7,8,9]. Focusing on the EEG technique, the electric potentials measured on the scalp reflect the neuronal activity and can be used to study a wide array of brain process in many different applications, such as BCI. Thanks to the great speed at which electric fields propagate, EEG signals have an excellent temporal resolution, but at the same time, they present some limitations related to:

- non-stationarity, which is the reason why learning models trained on a temporally limited amount of data, might generalize poorly with respect to data recorded at a different time on the same individual;
- high inter-subject variability due to physiological artifacts differences between individuals. This aspect can severely affect the performance of learning models;
- data collection, time-consuming, and restricted. Medical data is not usually available due to personal data regulation.

To solve these problems, time-consuming processing pipelines with domain-specific approaches are often used to clean, extract relevant features and classify EEG data. Removal of artifacts may be crucial to achieve good decoding performance. Consequently, some studies attempted to only apply minimal preprocessing such as removing or interpolating bad channels and leave the burden of learning from a potentially noisy signal on the neural network to extract true brain activity from the recorded signals to be correctly interpreted [10,11,12].

In this context, Artificial Intelligence (AI) provides a set of general approaches that models intelligent behavior with minimal human intervention with a great help in processing neural signals from the brain, including feature extraction and classification [13]. As a branch of AI, Machine Learning (ML) tools are often used to automate, extend, and improve EEG data analysis with the final aim of partially or completely solving the above-mentioned issues. Indeed, BCI systems are based, in many applications, on decoding pipelines that use extensively different machine learning algorithms. Before the deep learning (DL) revolution, the standard pipeline to analyze the EEG data combined techniques from signal processing and ML to enhance the SNR, dealing with EEG artifacts, extract features, and interpreting or decoding signals. DL is part of the field of machine learning methods based on artificial neural networks with the ability to use techniques that allows a system to automatically detect and classify features from raw data. DL models are deeper variants of ANNs with multiple layers, whether linear or non-linear.

Artificial Neural Networks (ANNs) aim to simulate intelligent behavior by mimicking the way that biological neural networks function [14]. The simplest artificial neural network is a single-layer architecture, which is composed of an input layer and an output layer that usually obtains poor performances in complicated data patterns [15]. In order to overcome this limitation and to improve the obtained performance, two kinds of neural network models were proposed: the Multi-Layer Perceptron (MLP) referred to as a Feed-Forward Neural Network (FFNN), which includes a so-call hidden layer between the input layer and the output layer and the Convolutional Neural Networks (CNNs), a natural extension to MLP, and thus applied in this context. Unlike MLPs, CNN architectures require computationally expensive operations, but they are appreciated to automatically extract relevant features instead of manual extraction techniques from high dimensional datasets [16]. CNNs are a sequence of layers, and each layer of the CNN transforms one volume of activations to another through a differentiable function. Autoencoders (AEs) are also often used: they earn the latent representations of input data (called encode) in an unsupervised manner and then use these representations to reconstruct output data (called decode) and Recurrent Neural Networks (RNNs), an extension of an FFNN, which is able to learn features and long-term dependencies from sequential and time-series data. Unfortunately, most of the existing machine learning studies focus on static data and cannot classify the dynamic changes of brain signals accurately for practical uses. This aspect requires novel learning methods to deal with dynamic data streams in BCI systems [17].

The diffusion of DL approaches has changed machine learning in many domains (e.g., computer vision, speech recognition, etc.) by providing general purpose and flexible models that can work with raw data to directly learn features and to capture the structure of data in an efficient and adaptable way for many different tasks.

Recent advancements in DL frameworks, based on Deep Neural Networks (DNN), drastically improve accuracy in image recognition, natural language processing and other applications. DNN is the extension of a standard neural network with multiple hidden layers, which allows the model to learn more complex representations of the input data. The key advantage of DL is a systematic approach of training groups of DNN layers, including unsupervised training of auto-encoders for hierarchical representation of raw input data (i.e., automatic feature selection and dimensionality reduction) and supervised re-training of several final layers in the transfer learning that compensate for data incompleteness. Deep learning works directly on raw brain signals, thus avoiding the time-consuming preprocessing and feature extraction, so deep neural networks can capture both representative high-level features and latent dependencies through deep structures [17]. Finally, one of the most important motivations for using deep learning on EEG processing is automatic feature learning [18].

DNN-based DL frameworks combine ultimate flexibility for data modeling with hierarchical representations, unsupervised pre-training, transfer learning and overall layer-by-layer training, which are all crucial for the discovery of viable models, even when data are incomplete and very complex. However, DNNs training could be very challenging due to a large number of data and hyper-parameters, ranging from the training algorithm parameters such as learning rate, neural network topology, number of layers and the number of nodes in each layer. It is extremely computationally expensive to train and more importantly to determine the training method and the hyperparameters for deep learning, which is still user dependent [18].

Among the different types of DL, such as unsupervised deep models or generative learning, the hybrid Deep Learning (hDL) combines both generative and discriminative models, which is the most used for human action recognition [19]. hDL was inspired by the further problems introduced by BCI and many of them were resolved through the use of action bank features [20]. hDL is often designed by the fusion of homogeneous CNNs and by the combination of those with other neural networks, such as RNN, Stacked AutoEnconders (SAEs) and others.

**Figure 1:**
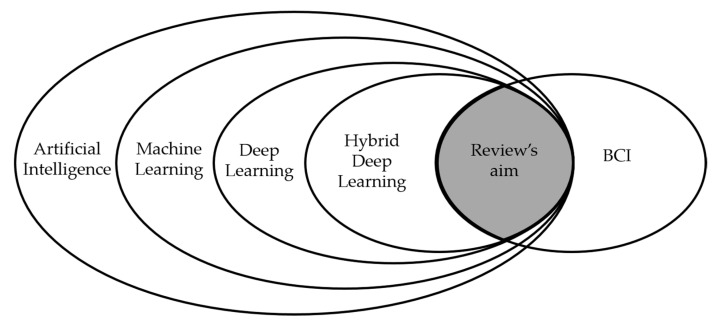
Schematic representation of the review’s aim.

With this review, we provide an overview of hDL-based BCI of the papers published in the last five years, since no papers were found before 2015 on this topic (Figure 1). A list of acronyms is reported in Appendix A—Table A1.

We have also reported methodological details about the various steps of the pipeline implemented for the different approaches, in order to give an idea of the most adopted techniques and processing steps.

We have reported that different choices need to be considered when handling hDL-based BCI. In particular, a careful choice needs to be made on the methodology used for detecting mental tasks. Portable and non-invasive methodologies should be preferred, such as EEG, Magnetoencephalography (MEG) or functional Magnetic Resonance Imaging (fMRI). Among them, EEG alone was the most used in the revised papers, with 93.62% of the case (44/47 papers) probably due to the low cost of the EEG system and the simple way to record the brain signal (EEG in combination with other modalities reaching 100% of the time). However, despite its advantages, such as portability, low cost and non-invasivity, EEG needs strong data preprocessing such as data-filtering and channel interpolation among other more advanced preprocessing methods such as Independent Component Analysis (ICA) used to reduce biological and non-biological artifacts [6,7,8,9,21].

In this respect, 78.72% of the reviewed papers (37/47) used some of the above-mentioned preprocessing methods. Once the recording technique is decided upon, it is necessary to focus on the task to be implemented in order to capture the mental task, since BCI is a system that should establish a direct communication pathway between the users’ brain activity (mainly people disabled by neuromuscular disorders such as amyotrophic lateral sclerosis, cerebral palsy, stroke, or spinal cord injury) and external effectors [22]. Motor Imagery (MI) seems to be the most used task in the reviewed papers, being used 55.32% of the time. After these considerations, it is necessary to identify the best features to be extracted from the data

To answer these questions, our review is organized as follows: an extensive description of the analyzed papers has been presented in Section 2, summarizing the relevant information for the proposed approaches and how the papers were selected and assessed. The results of the study have been reported in Section 3, grouped in the main steps of a standard pipeline, particularly focusing on the hybrid deep learning architecture, introducing applications and datasets. Section 4 introduces the discussions and Section 5 introduces the possible future studies.

## 2. Materials and Methods

**Figure 2:**
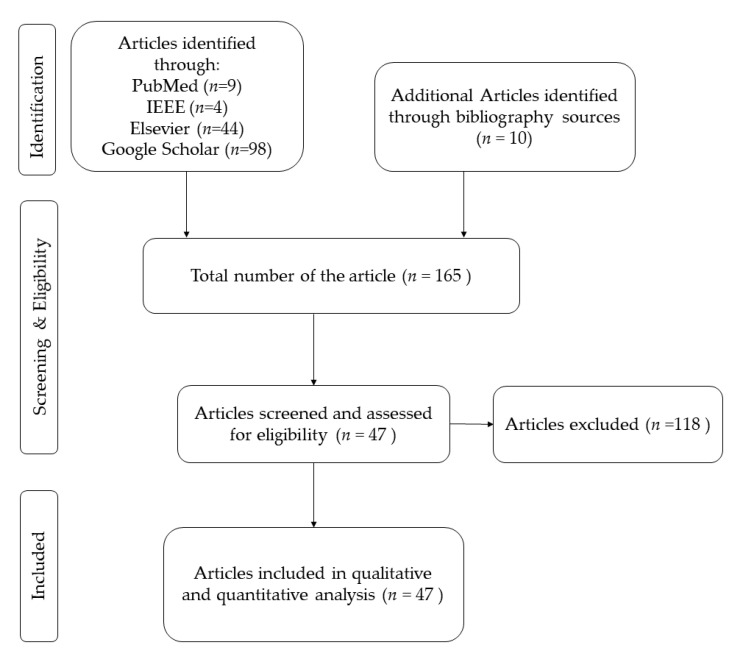
Flowchart of the selection process of the papers.

English papers, including full articles, were selected for the review. To collect data from a variety of resources, four academic research databases were used: Google Scholar (https://scholar.google.com/), PubMed (https://pubmed.ncbi.nlm.nih.gov/), IEEE Xplore (https://ieeexplore.ieee.org/Xplore/home.jsp), and Elsevier Science Direct (https://www.sciencedirect.com/) using the following queries BCI + “Hybrid Deep Learning”; BCI AND “Hybrid Deep Learning”; BCI Hybrid Deep Learning; and BCI + “Hybrid Deep Learning”, respectively, for Google Scholar, PubMed, IEEE Xplore, and Elsevier Science Direct. We have applied the Journal filter in IEEE Xplore and Research Articles for Engineering in Elsevier Science Direct. Google scholar query produced 98 papers, while PubMed query produced 9 papers, IEEE Xplore produced 4 papers, and Elsevier Science Direct produced 44 papers. The last query in all the databases was done on the 13th of November 2020. The overall number of collected papers was 155 with 10 papers added to the list from the literature review (165 papers in total). Papers that did not use a hybrid algorithm or were not in the field of BCI were eliminated from the original 165 papers list. Duplicated papers (i.e., papers that were found in more than one database) and reviews were eliminated as well. The resulting list of papers consisted of 47 papers (see also Table A2 in Appendix B). Figure 2 shows the flowchart for building the database considered in this review, which consists of 47 original papers ranging from 2015 to 2020, which uses hDL algorithms in BCI systems. There were no papers found before 2015: this is because the hybrid deep learning methodology was applied to BCI for the first time in 2015 with the two seminal studies [12,23].

## 3. Results

### 3.1. Brain Intention Recordings

EEG is widely used in Brain-Computer Interfaces [24], as is highlighted by the 47 articles reviewed, here in which all of them have only used EEG, except three papers that used EEG combined with EOG [4], EEG with ElectroOculoGraphy (EOG), ElectroMyoGraphy (EMG), Skin Temperature (ST), Galvanic Skin Response (GSR), Blood Volume Pressure (BVP), Respiration Signal (RS) [25] and EEG plus EOG [26]. While EEG has proven to be a crucial tool in many domains, including BCI, it still suffers from some limitations that hamper its effectiveness due to its long pre and post-processing. In this context, DL [5] was introduced with the goal of simplifying the long pre and post-processing steps, which was most of the time also user-dependent, employing its automatic end-to-end learning of preprocessing, feature extraction, and classification modules, while also reaching competitive performance on the target task. This high expectation was supported by the enormous success obtained by DL in processing complex data such as images, text, and audio signals [27]. However, the same success seems to be far away in the context of EEG based BCI. The main reason for that might be attributed to EEG peculiarities, such as low SNR [5], which makes EEG data different from images, text, and speech data. Therefore, the architectures and practices that are currently used in DL on other types of data may not be simply moved to the EEG data. This was also supported by the results obtained in our review where we have found that only 21.28% of the papers (10/47) did not use any preprocessing or they did not declare any preprocessing step (N/A in Table A2—Appendix B, “Pre-processing” column). Among the remaining 78.72% of the papers (37/47), at least a bandpass filter or more advanced preprocessing methods, or even a combination of the two, has been applied, as detailed in the section below.

### 3.2. Preprocessing of the Data

Since preprocessing seems to still be an important step that cannot be simply bypassed by DL architecture, we divided the papers reviewed into three main categories: (i) No preprocessing applied (N/A); (ii) Basic preprocessing such as filtering; and (iii) Advanced preprocessing such as Blind Source Separation (BSS) methods or semi-BSS [28,29], as in the case of wavelet-enhanced Independent Component Analysis (wICA) [30]. Among the 47 papers, 21.28% did not apply any preprocessing step, 61.7% applied basic preprocessing, consisting mainly in Band-Pass Filter (BPF), and 17.02% applied a more advanced BSS approach such as ICA or Principal Component Analysis (PCA) or Denoising Autoencoder (DAE) or Common Spatial Pattern (CSP) in combination with BPF. However, since none of the papers reviewed here compared the performance base on the presence and absence of the preprocessing, we were not able to properly investigate whether the presence or the absence of the preprocessing step can increase the accuracy performance. On this particular aspect, studies that directly investigate this issue are more than welcome.

### 3.3. Normalization of the Data

**Figure 3:**
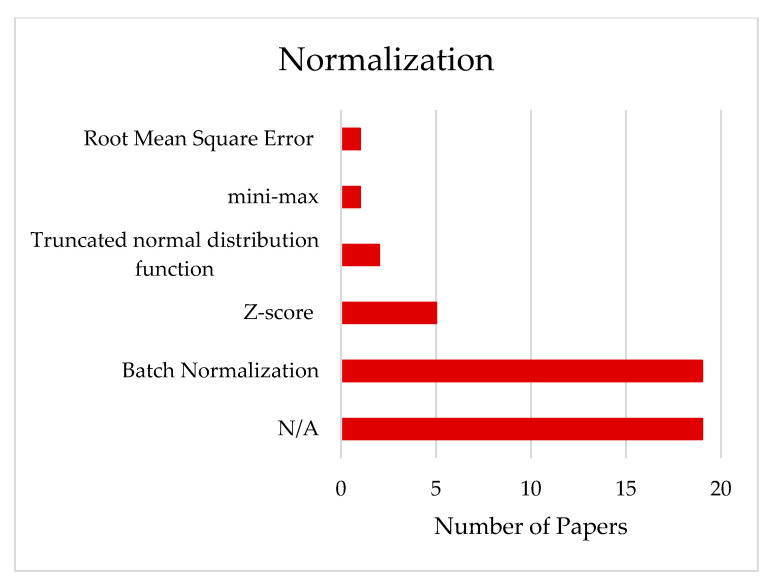
Distribution of the normalization methods across the reviewed papers.

Normalization of the data is the set of pre-processing steps aimed at eliminating information redundancy and inconsistency from the database to control the complexity of the neural network and to obtain performances that can be generalized for several fields of application [31]. As can be seen from Appendix B—Table A2 and Figure 3, different kinds of normalizations are applied in 59.57% of the cases (28 papers on 47). Among the normalization methods, Batch Normalization (BN) is the most used, at 67.85% of the time, followed by Z-score, used 17.85% of the time, Root Mean Square Error (RMSE) in 3.58%, min/max Normalization in 3.58% of time, and Truncate normalization distributed function, in 7.14% of the time. BN is a method used to make artificial neural networks faster and more stable through the normalization of the input layer by re-centering and re-scaling [32]. The major benefit of BN is the training speed up of deep neural networks by reducing the internal covariance shift, which is “the change in the distributions of internal nodes of a deep network” [32,33]. It also acts as a regulator, in some cases eliminating the need for dropout and consequently avoiding overfitting [34].

### 3.4. Features Extraction

Generally, the BCI system is considered to be a pattern recognition problem, where the main two tasks for the BCI system are feature extraction and classification. The features are a set of information that represents the main characteristics of the data in hand. Those features are used as input for the classifier to perform the pattern recognition task, translating the mental state in information for BCI [35,36,37]. Feature extraction is a very sensitive step in the BCI system since reduces the data into a limited number of data that should accurately represent the full data, which has a tremendous effect on the efficiency of the classification phase. Choosing the most significant features is important to achieve high recognition performance [7,38]. Features are normally extracted using statistical and signal processing tools. Lately, thanks to the advent of deep learning, feature extraction is done automatically by the chosen architecture. For example, CNN takes a 2-D matrix as input and automatically extracts hidden features using spatial filters [39].

Tang and colleagues [40] used the Short-Time Fourier Transform (STFT) as a 2-dimensional EEG representation as input for the feature extraction step done by CNN. Similarly, Dai and colleagues [41] used a time-frequency domain representation (Spectrogram image) of the EEG obtained via the STFT. In Jingxia and colleagues [42], frequency-domain features were also used. They extracted 64 Power Spectral Density (PSD) features by using Hamming window with a width of 0.5 s in 1–47 Hz frequency. Another direction was taken by Maryanovsky and colleagues [43] towards statistical features like variance.

**Figure 4:**
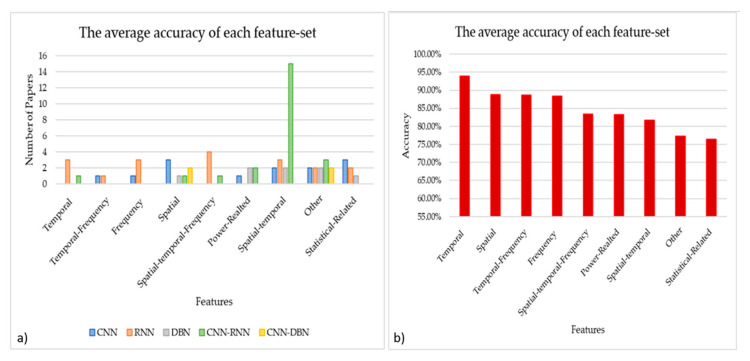
(**a**) Feature extraction distribution across architectures. (**b**) The accuracy obtained for each feature. Note that bar “Other” grouped the following features extracted by Selective Attention Mechanism (SAM), Optical Flow from the EEG video, 425 silent physiological features from the 7 signals, and Hilbert–Huang spectrum (HHS), High-level features, Linear domain features (Autoregressive coefficient), and Non-Linear domain features (Approximate entropy, Hurst Exponent).

As we have seen in the reviewed studies, we can cluster the features extraction into nine groups, with their respective usage percentages: spatial features (10.61%), temporal features (6.06%), frequency features (6.06%), temporal-frequency features (3.03%), spatial-temporal features (33.33%), spatial-temporal-frequency features (7.58%) power-related features (7.58%), statistical-related features (9.09%), and another group of features that are not related to any of the previous groups (16.67%). These data are shown in Figure 4a, through which it is possible to notice the dominance in the use of the CNN-RNN hybrid architecture for the automatic extraction of spatial-temporal features. Moreover, it is possible to see how CNN-based architectures are used to extract the different categories of features, except for the temporal ones, for which the use of RNN-based architectures is preferred. Besides, RNN-based architectures are not used for both spatial and power-related features. Based on these results, we can say that temporal features achieved the best results for the mean (93.94%), followed by spatial features (88.73%). Interestingly, the spatial-temporal features were used by most of the studies achieved (81.63%), as illustrated in Figure 4b.

### 3.5. Hybrid Deep Learning Architecture

**Figure 5:**
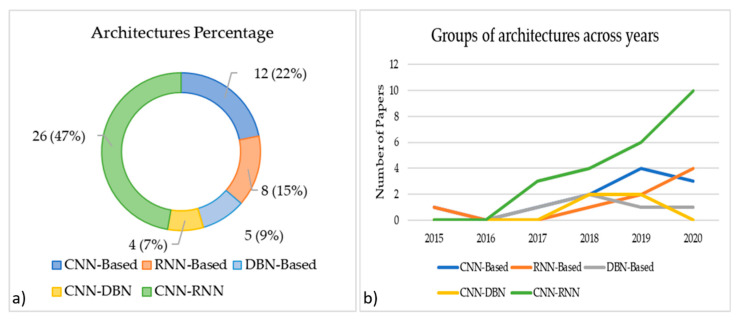
(**a**) Architecture percentage distribution. Note that there are papers that used more than one network; (**b**) the trend of architectures used across the years.

In the last years, many types of architectures were developed. Each architecture has its special characteristics regarding a field of information. By merging different kinds of networks, we can extract deeper features than using the deep learning algorithm alone [44] (see Appendix C for a more detailed overview of Deep Learning). Thus, the choice of hDL architecture becomes an important point in the hDL pipeline. Figure 5a shows the percentage of the studies that used different hDL architectures. CNN-RNN is the most used architecture with 47% of the cases. It combines the spatial features extracted from CNN and temporal features extracted from RNN. CNN-based architecture instead uses spatial features than temporal ones and is the second choice on the reviewed papers (it is chosen in 22% of the case). The other architectures are DBN-based, chosen in 9% of the cases, RNN-based chosen on 15% of the cases and CNN-DBN was chosen in 7% of the cases.

In Figure 5b, it is provided with the distribution of the different architectures used across the years. To the best of our knowledge, two seminal studies [12,23] in 2015 introduced the hDL-based BCI using CNN-based and RNN-based, respectively. The success of CNN might be due to its capabilities to extract spatial information from images (2D input) in a hierarchical structure as it showed great success in the computer vision field [45]. However, from 2017, a modified version of the CNN architecture seems to be predominantly used in the field, i.e., CNN-RNN with a constant increase in its presence in the studies ranging from 2017 until 2020 with three papers in 2017 (i.e., 60% of the case), four papers in 2018 (i.e., 36.36% of the case), six papers in 2019 (i.e., 40% of the case) and 10 papers in 2020 (i.e., 55.55% of the case). Despite the advents across these years of other types of architectures such as DBN [46], and a combination of CNN and DBN named CNN-DBN [41]. Figure 5b also clearly shows an increasing trend in the last five years in the use of hDL-based BCI. Interestingly, in 2020, the number of papers using CNN or a combination of it with RNN or DBN was 72.20% (i.e., 13 papers on 18), with a performance (mean ± standard deviation) of 82.54 ± 6.04% in the case of the MI task, 94.74 ± 4.62% in the case of the SSEP task.

**Figure 6:**
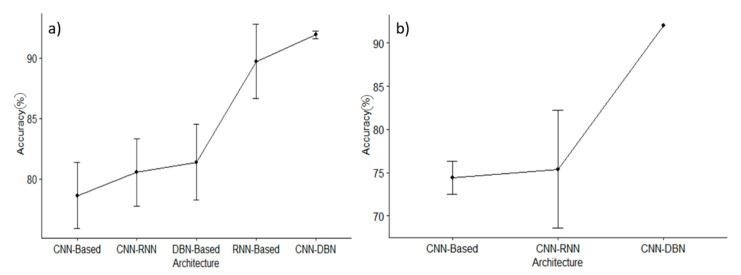
Average accuracy ± standard deviation for each architecture; (**a**) all datasets; (**b**) BCI Competition IV dataset.

Figure 6a shows the accuracy with respect to the different architectures. The standard deviation and the mean are evaluated by considering all data and it is worth noting that the best results are achieved, considering CNN-DBN-based architectures, whereas the lowest performances in terms of average accuracy are the CNN-based architecture. Since BCI Competition IV is considered to be a benchmark to test hDNN approaches, the same analysis is only reported for this benchmark in Figure 6b. In this case, only one sample is related to the CNN-DBN-based architecture.

### 3.6. Optimization

Optimization is one of the fundamental steps of machine learning. The idea behind most machine learning algorithms is to build an optimization model and to set-up the parameters throughout the training session. As can be guessed, there are several ways to approach this step; however, the best way to proceed is still an open research question in the deep learning literature [47]. The difficulty to find the optimal solution lies in searching the balance between the minimization of the cost function and the performance, which in turn minimizes the difference between the training error and the actual error obtained from the test set (i.e., the training set). It becomes clear that the obtained results strictly depend on the choice made in this step.

**Figure 7:**
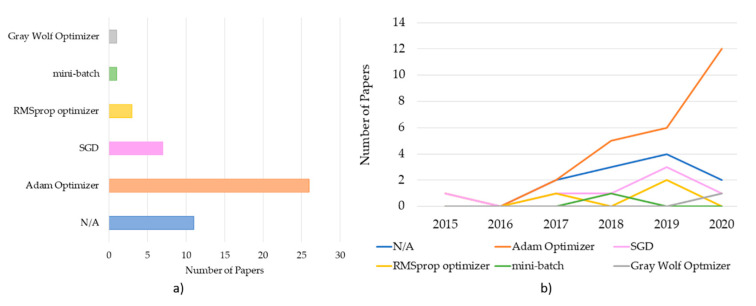
(**a**) Optimization algorithms distribution across the reviewed papers. (**b**) Trend of Optimizers across years (note that N/A refers to the papers that did not state the use of the optimizer).

While this step is a crucial step for achieving good results, in 23.40% of the papers; however, they did not report the used optimization algorithm (see Figure 7a). From the remaining 76.6% that declared that the optimization algorithm was used, the most used was the ADAptive Momentum (ADAM) optimizer that was used in 55.3% of the cases.

ADAM estimation is an advanced Stochastic Gradient Descendent (SGD) method, which combines adaptive methods and the momentum method [48]. It uses first-order momentum estimation and second-order gradient estimation to dynamically adjust the learning speed of each parameter; it also adds bias correction. ADAM is very stable in practice, and it is suitable for most non-convex optimization problems with large data sets and high dimensional space [47]. Despite its massive use, the algorithm may not converge in some cases. After the ADAM optimizer, the other optimization algorithm mostly used is the SGD in 14.89% of the cases. The SGD [47] is an iterative method for optimizing an objective function with suitable smoothness properties. The biggest advantage of using these methods with respect to other methods rely on the fact that the calculation time for each update does not depend on the total number of training samples. The calculation could be significantly sped up by removing the computational redundancy [47]. However, the main limitation of SGD is choosing the optimal learning rate. To do so, the trial and error method is suggested, since there is no predefined standard [49]. Among the other optimizer methods, the most relevant is the SGD, used in 14.89% of the papers, Root Mean Square Propagation (RMSProp) used 6.38% of the time, mini-batch used 2.30% of the time, and Gray Wolf Optimizer (GWO) used 2.30% of the time. To be noted, as reported in Figure 7b, the ADAM algorithm steadily increased across years, showing that the community more and more often uses that algorithm.

### 3.7. Number of Layers

**Figure 8:**
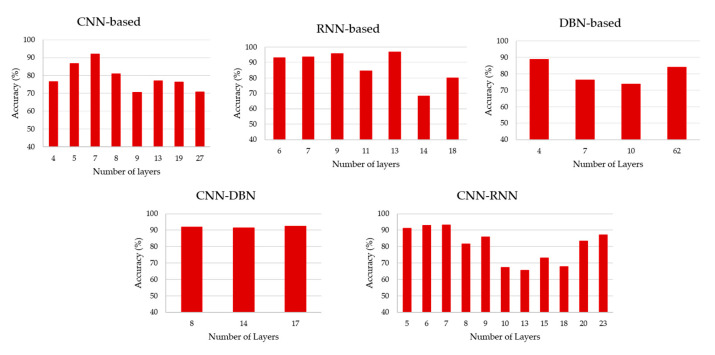
Number of layers vs. accuracy for each architecture. Note that some papers used more than one network with a different number of layers.

The adjective “deep” in deep learning refers to the number of layers through which the data are transformed from the first layer to the second one and so on in a hierarchical fashion [27]. Despite the adjective “deep” in deep neuronal networks, which might be induce the idea of a large number of layers in the architecture, this is not always the case. Here, we reviewed this aspect and showed that the number of layers is lower or equal to 10 in 50.90% of the cases and just 16.36% higher than 20 layers; this matches what Roy and colleagues also reported [18]. In Figure 8, we showed the accuracy in respect to the number of layers, for each architecture. Regarding the studies that proposed different architectures, we only considered the number of networks. From Figure 8 and in Appendix B—Table A2, our results support Roy and colleagues’ results [18] that there is no standard procedure to choose the number of layers, since the choice depends on many factors, such as the data in hand, which was used as an input, the type of task to be performed, hyperparameters tuning, etc. A Person’s correlation test was performed between the number of layers and the performance of each architecture separately to test if the increasing number of layers corresponds to an increase in accuracy (CNN-based: R = −0.53; *p*-value = 0.089, RNN-based: R = −0.51; *p*-value = 0.16, DBN-based: R = 0.28; *p*-value = 0.65, DBN-CNN: R = 0.16; *p*-value = 0.89, and CNN-RNN: R = −0.21; *p*-value = 0.28).

### 3.8. Application, Datasets and Task/Protocol

**Figure 9:**
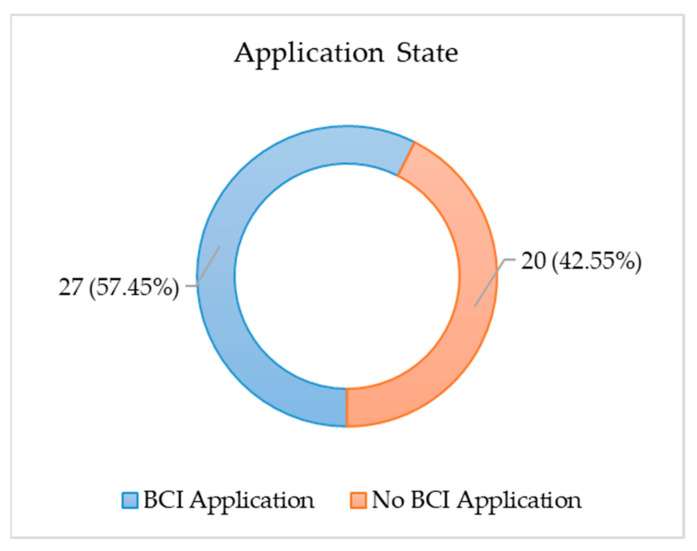
Specified vs. not specified BCI application.

Figure 9 shows that 57.45% of the reviewed papers have a specific BCI application, such as medical care, communication, mental state detection, person identification, emotion recognition, motor imagery recognition, and data augmentation. The remaining papers do not have a specific application: this category aims to develop the classification algorithm and to evaluate its performance, regardless of the application by tackling the challenges faced by BCI with respect to the accuracy of classification and precision. In other words, those studies were conducted more for classification and accuracy than for applicable BCI.

**Figure 10:**
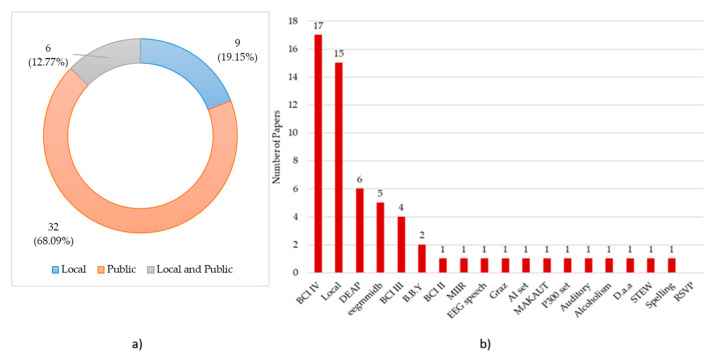
(**a**) Percentage distribution of the dataset type. (**b**) Distribution of Datasets across the number of papers. Note: Some papers used more than one public dataset to compare the performance of their model. AI set: AI Dataset; Alcoholism: Examining EEG-Alcoholism Correlation; Auditory: auditory multi-class BCI; B.B.Y: Bashivan, Bidelman, Yeasin EEG data set; BCI II: BCI competition II; BCI III: BCI competition III; BCI IV: BCI competition IV; DEAP: DEAP dataset; D.a.a: Decoding auditory attention; EEG speech: EEG based speech dataset; eegmmidb: Physionet eegmmidb; Graz: Graz University Dataset; Local: Local Dataset; MAKAUT: MAKAUT Dataset; MIIR: OpenMIIR; P300 set: Exploiting P300 Amplitude changes; STEW: “STEW” dataset.

Figure 10a shows the datasets used in the reviewed papers. We have classified the papers into three classes: papers that used a public dataset (68.09%), such as BCI competitions datasets, papers that used their dataset (19.15%), named local datasets, and papers that used both public and local datasets (12.77%). In Figure 10b, how the databases were used among the paper reviewed is illustrated. BCI-competition IV was used 17/47 times, which makes it the most used public dataset. This might refer to the reliability and flexibility of this dataset. Database for Emotion Analysis using Physiological Signals (DEAP) was used by six papers, while both Physionet eegmmidb (EEG Motor Movement/Imagery Dataset) and BCI competition III were used in five and four papers, respectively. Bashivan, Bidelman, Yeasin EEG data set was used twice, while the other datasets were only used once.

**Figure 11:**
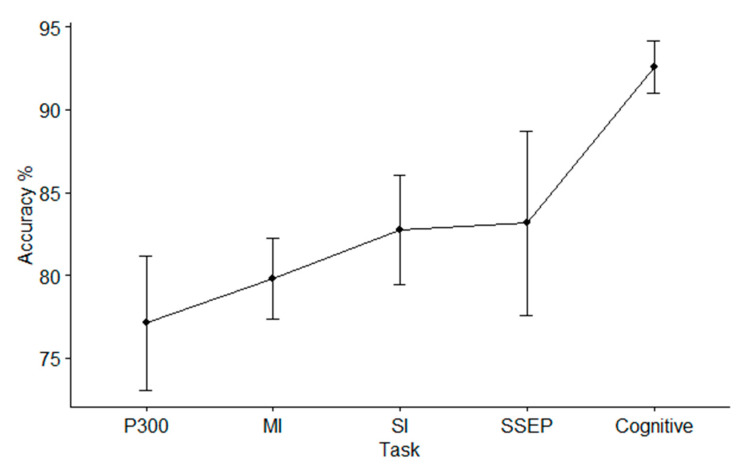
Mean ± standard deviation accuracy across tasks.

Figure 11 shows the accuracy with respect to the tasks, where the task SSAEP has been removed from the analysis, since only one paper has used it. Mean ± standard deviation was evaluated by considering all datasets. The best accuracy level was achieved during a cognitive task.

### 3.9. Hybrid Deep Learning (hDL) Performance

**Figure 12:**
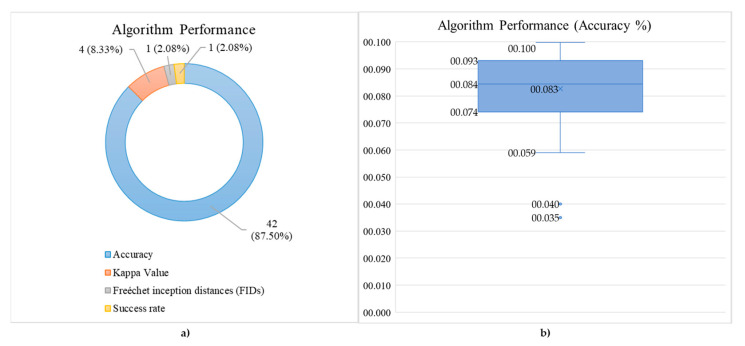
(**a**) Percentage distribution of the performance estimated by different algorithms. (**b**) Classification percentage accuracy.

The hDL performance has been and was measured using different metrics (see Figure 12a): (i) classification accuracy was the most used (87.50% of the time); (ii) Kappa value, which was used 8.33% of the time, indicates the agreement of the evaluated classification with respect to different studies in the same conditions. In other words, it measures the inter-rater reliability that can be considered as a score of consistency given by the same dataset/subjects across different architectures [50]; (iii) Freéchet Inception Distances (FIDs), which measures similarity between augmented EEG data and real EEG data [51], which was used 2.08% of the time, as well as the success rate. Concerning the accuracy, the box and whisker chart has also been shown in Figure 12b. It shows information about the statistical quartiles (74.15%, 93.10%), median (84.45%), mean (82.65%), the maximum (99.74%), and the minimum (59.00%). Two networks were treated as outliers since it suffers from very low accuracy (40.00% and 35.00%). The average accuracy ± standard deviation is 82.6 ±14.18%.

**Figure 13:**
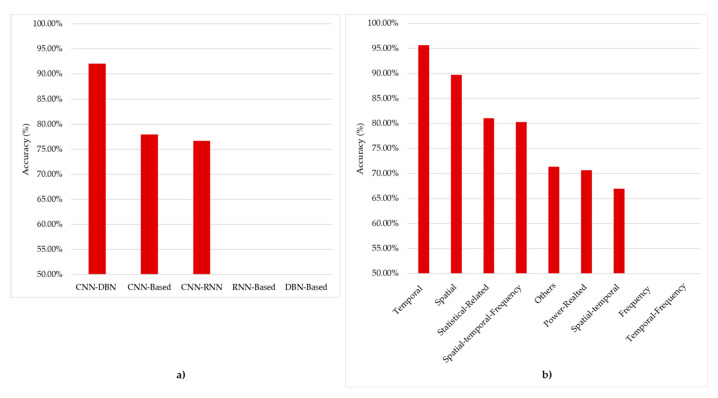
Accuracy across hDL architectures and features on the BCI-Competition IV subset. (**a**) Accuracy of the architectures. (**b**) Accuracy of the extracted features.

To better test the accuracy performance among hDL architectures and the accuracy obtained across the different features, we have calculated those metrics on the same dataset (i.e., the BCI competition IV dataset, the most used in the reviewed papers). We have found that the best accuracy was obtained by CNN-DBN (92.00%), which was only used in one study with BCI competition IV dataset, while CNN-based and CNN-RNN achieved 77.88% and 76.63%, respectively (Figure 13a). Temporal features reported the best performance, 95.62%, which was achieved by using the CNN-RNN architecture, while spatial features reached 89.68%; CNN-RNN also achieved this (see Figure 13b). Notably, the results obtained in Figure 13b (i.e., results obtained for the BCI IV dataset using MI task) follows the trend of the results obtained for all the datasets shown in Figure 5b. However, this comparison might not be very accurate, since only two studies use CNN-DBN in comparison to three papers in CNN-based, and five papers in CNN-RNN. Therefore, more studies regarding the CNN-DBN are encouraged to be conducted since it shows promising results. There are no studies that used frequency features or temporal-frequency features extracted from BCI-competition IV. Additionally, no papers studied the RNN-Based or DBN-Based in the same database. This opens the door to making more hDL combinations.

## 4. Discussion

In this work, we have reviewed the main results published from the seminal studies in 2015 [12,23] to the end of 2020, with the aim of elucidating the main aspects of hDL-based BCI. Our goal was to give an overview of the hDL architecture that was the most used in BCI. We have also given information about the trends across the years regarding the hDL-based BCI. Our challenge was to provide a guide on the choice to be made when an hDL-based BCI approach is implemented, based on the choices that have been made in the last five years in this field.

### 4.1. Preprocessing

One of the main reasons for using hDL is the growing trend to use raw EEG data directly as an input of the hDL without external preprocessing and feature extraction. Even though preprocessing is a very important step in the BCI system and physiological signals analysis. Some efforts have been made to automate preprocessing [6,9,52,53] and this could be a step towards BCI systems [54,55,56]. We expect this automatization, which goes beyond the use of hDL, to gain popularity as a replacement for traditional processing pipelines. In this respect, we have shown that only 21.28% of the papers did not use any type of pre-processing, despite the use of hDL architectures. This trend was also highlighted by Roy and colleagues [18]. The use or not use of preprocessing before hDL-based BCI is still under debate, since the performance obtained is not clearly in favor of one of the two. For example, some papers [10,11,57] obtained good performance, 98.81%, 95.33% and 92%, respectively, even though they did not use any preprocessing step. However, Jeong and colleagues and Saidutta and colleagues [26,58], using automated and advanced preprocessing, reached a performance of 87% and 81%, respectively.

One point to take into account is the shape of the input used for the hDL; most of the papers used a matrix as input for CNN. This is not an unexpected result since CNN was designed to classify RGB images, and usually, most frameworks for deep neural networks present examples of CNN 2D convolutional. Some papers used a matrix of raw EEG signals (signals in rows, and channels in columns) as a 2D input for CNN, while Dai and Colleagues [41] transformed EEG into spectrogram images and used it as a 2D input to the CNN using Short Time Fourier Transform (STFT). Others, such as Chuanqi and colleagues and Tan and colleagues, [20,59], prepared their data as a sequence of images to create an EEG video where each frame is an image and each pixel represents a channel location. The color of each pixel refers to an extracted feature, for example, PSD. However, among the papers that have transformed the EEG data into images and have used the same architecture, but with different preprocessing types, the paper that used advanced preprocessing data [20] achieved higher accuracy (72.22%) concerning the one without preprocessing [59], which only achieved 35% accuracy. In the latter case, however, different datasets were used, which means we cannot decide whether the difference in the accuracy is due to the dataset or the preprocessing. What we can say is that the input shape might determine whether or not the preprocessing is needed and at the same time, which kind of hDL architecture is the more appropriate for the data in hand.

To shed new light on this point, we have investigated the papers that have used the same datasets, in particular the BCI competition IV used in 36.1% (17/47) of the papers. In this subset of the revised papers, only one study [60] avoided any type of preprocessing, reaching a performance of 59%. The others that used preprocessing reached 74.58% accuracy on average. Based on this, we can conclude that it is advisable to preprocess the data, even though the hDL framework is used.

### 4.2. hDL Framework

#### 4.2.1. Feature Extraction

Referring to the papers inspected in this review, we can observe that the temporal features have obtained the best performance (93.94%). Additionally, spatial (88.73%) and temporal-frequency (88.71%) features also have good performance. We can associate these results with the intrinsic nature of the EEG data and its high temporal resolution. On the contrary, frequency features reached a lower accuracy performance of 88.36%. We can also observe that by merging spatial and temporal features, the mean accuracy was reduced to 81.63% with respect to the 93.94% obtained using only temporal features. We are also aware that this comparison is not very accurate because of the lack of data, since temporal features and spatial features were used by four and seven papers, respectively, while spatial-temporal features were used in 22 papers. This encourages more exploration toward temporal features and spatial features separately.

#### 4.2.2. Normalization

It is worth noting, from Figure 4, that there is an increasing trend toward the use of Batch Normalization (BN) among other algorithms. While BN is the most used, 67.85%, there is still a lack of understanding of its working mechanism. This debate is carried between some researchers who claim that the internal covariate shift is not reduced significantly by batch normalization, despite common belief [33]. Others argue for attributing the good performance to smoothing the objective function, while others propose that length-direction decoupling is the reason behind its effectiveness [61,62]. From what has been observed, the architecture that used BN did not suffer from weak performance; therefore, BN is encouraged to be used in hDL-based BCI, since it was tested in the majority of papers.

#### 4.2.3. Architecture

As seen in Figure 6, CNN-RNN and CNN-based are the most used architectures (47% and 22%, respectively), CNN is known to work well when there is a spatial relationship between the input data. This characteristic seems to be counterintuitive for the EEG data. Instead, RNN performs well with sequences of data, like time-series that are more suitable for the EEG characteristics and its high temporal resolution. Combining, both architectures (i.e., CNN and RNN), we merge the spatial and temporal characteristics of the EEG as well. By comparing CNN-based and RNN-based with DBN-based, we can see that DBN has been used less often in the hDL-based BCI system, despite DBN being a good choice when continuous values are presented as an input that looks perfect for EEG data. However, it does not benefit from any data spatial relationship [63]. From the architectural point of view, this DBN lack has been recovered by merging it with CNN. However, this choice has been adopted in only 7% of cases, and so more investigation attempts are encouraged using CNN-DBN architecture.

As we have seen, CNN alone or in combination with other architectures are the most used. We believe that this is more because of the fact that CNN is considered an automated feature extractor with respect to its ability to handle spatial information, at least for the EEG data. The ability to extract features automatically is due to its embedded image filters implemented on CNN.

We could summarize that hDL is a subset of machine learning that uses a complex combination of layers [27]. The main advantage of hDL in respect to ML is less needed for human intervention [17]. However, the cost of this advantage could be summarized in two steps: the need for larger training sets [43] and the high computational efforts required [64,65,66].

#### 4.2.4. Number of Layers

There is no specific rule to decide the number of layers. Generally, the main goal is to minimize the number of layers to as minimum as possible to reduce the required computational efforts. However, the trials and error approach is the most used to decide the number of layers [67]. Some claims say that number of layers should be lower than the number of the inputs [67]. From what we have found, there is no relationship between the number of layers and the performance. Therefore, it is encouraged to reduce the number of layers with the aim to reduce computational time cost. From the papers reviewed here, we did not find any relationship between the number of layers and the accuracy performance, suggesting that a tradeoff between accuracy performance and computational time by trial-and-error approach is recommendable. Our study does not cover the effect of the number of neurons in each layer, which will be an interesting topic to explore.

#### 4.2.5. Optimization

From the reviewed papers, it is noticeable that ADAM is more desirable to be used in hDL-based BCI systems. This is due to its stability in comparison to other optimization methods. Empirically, it was shown that ADAM outperformed other optimizers in hDL-based BCI systems [68,69].

Generally, optimizers perform better on preprocessed EEG data since they have a higher signal-to-noise ratio. Instead, according to Kingma and colleagues [48], ADAM optimizer performs better than other optimizers with data that has low SNR. Based on that, it seems mandatory to use ADAM, especially in the case raw EEG data are used. Another reason why ADAM should be more attractive with respect to others is that it combines the advantages of other optimizers like AdaGrad and RMSProp [48].

## 5. Conclusions

In this review, we have highlighted the features necessary to develop a pipeline for hDL based BCI, starting from the seminal studies proposed in 2015. Our investigation revealed that electroencephalography is the most used signal to record human intentions. This choice, in our view, is more due to the comfort of using EEG and its low cost rather than a real choice based on the quality of the recorded data. In any case, the intrinsic EEG low signal-to-noise ratio requires the pre-processing of EEG data intending to increase the SNR, with a huge investment of time and dedicated expert personnel. Pre-processing of the data might be a characteristic in favor of using hDL architecture charging this aspect on the hDL architecture itself. Unfortunately, our results showed that, among the papers that did not use data pre-processing (about 79%), the accuracy of the results was lower, on the basis of the same architecture used. Furthermore, among those that have used data pre-processing, the works that have implemented advanced pre-processing methodologies, such as Blind Source Separation, are those that have obtained the best accuracy results. This trend was also observed by fixing the dataset used.

Another noteworthy observation concerns the features used. Time features appear to be the most effective with 93.94% accuracy. This aspect is in line with the EEG technique used, which is known to have a strong point in its temporal information. Finally, the most widely used architecture was the Convolutional Neural Network, combined with the Recurrent Neural Network, which combines the spatial (CNN) and temporal (RNN) characteristics of the EEG. In this case, the spatial characteristics refer to the time-frequency images generated, starting from the EEG data, and not the spatial accuracy in terms of localization of the electrical neuronal activity that is disreputably weak in EEG.

In conclusion, we can say that it is still advisable to pre-process the data, even if hDL architectures are used, and that the best architecture to be used strictly depends on the data in hand.

## 6. Open Challenges

Overall, the hDL-based BCI system is a promising framework due to its flexibility, reliability and high accuracy. However, this field is not fully explored and has many gaps that need to be bridged. As a conclusion of our review, we provide a list of open challenges:

1. More research is needed that uses other brain imaging techniques like functional Near-Infrared Spectroscopy (fNIRS), fMRI and MEG with the aim to investigate the richness of the information that the brain signal is able to bring.
2. Investigating the effect of the presence or absence of preprocessing on the data and the performance of hDL architecture.
3. Investigate the effects of the data’s input shape and their dimensionality.
4. Automating the entire pipeline of the hDL-based BCI system.
5. More exploration towards spatial and temporal features because it achieved high performance.
6. New architecture combinations are encouraged to be explored between frequency features and temporal-frequency features with RNN-based and DBN-based architectures.

## Appendix A

**Table A1:** Abbreviations.

Abbreviations	Meaning	Note
**AE**	Autoencoder	Artificial Neural Network
**AEP**	Azimuthal equidistant projection	Projecting Algorithm
**AI**	Artificial Intelligence	-
**ADAM**	ADAptive Momentum	Optimization Algorithm
**ALPS**	Age-Layered Population Structure	Genetic Algorithm
**BCI**	Brain-Computer Interface	-
**BGRU**	Bidirectional GRU	Recurrent Neural Network Structure
**BN**	Batch Normalization	Normalization Algorithm
**BPF**	Band Pass Filter	Signal Processing tool (Filter)
**BSF**	Band Stop Filter	Signal Processing tool (Filter)
**BSS**	Blind Source Separation	Signal Processing tool
**BVP**	Blood volume pressure	Physiological Signal
**CAR**	Common Average Reference	Signal Processing tool
**CCV**	Channel cross-covariance	Statistical Extracted Feature
**CNN**	Convolutional Neural Network	Deep Learning Neural Network
**CRAM**	Convolutional Recurrent Attention Model	Convolutional Recurrent Neural Network
**CSP**	Common Spatial Pattern	Signal Processing tool
**CSTP-NN**	Common Spatiotemporal Pattern Neural Network	Artificial Neural Network
**D-AE**	Denoising Autoencoder	Artificial Neural Network
**DBN**	Deep Belief Network	Deep Learning Neural Network
**DBN-GC**	Deep Belief Network Glia Cell	Deep Learning Neural Network
**DE**	Deferential Entropy	Extracted Feature
**DEAP**	Database for Emotion Analysis using Physiological Signals	Dataset Name
**DL**	Deep learning	-
**DNN**	Deep Neural Network	-
**DWT**	Discreet Wavelet Transformation	Signal Processing tool
**EEG**	Electroencephalography	Physiological Signal
**eegmmidb**	EEG Motor Movement/Imagery DataBase	Dataset Name
**ERP**	Event-Related Potential	Pattern in Electroencephalography
**MI**	Motor Imagery	Task/Protocol
**EMG**	Electromyography	Physiological Signal
**EOG**	Electrooculography	Physiological Signal
**FC**	Fully Connected	A layer in Deep learning Neural Network
**FBCSP**	Filter Bank Common Spatial Pattern	Signal Processing tool
**FIDs**	Freéchet inception distances	Evaluation metric
**FIR**	Finite Impulse Response	Signal Processing tool (Filter)
**fNIRS**	Functional Near Infra-red signal	Physiological Signal
**GA**	Genetic Algorithm	Artificial Intelligence Algorithm
**GRU**	Gated recurrent unit	Recurrent Neural Network Structure
**GWO**	Gray Wolf Optimizer	Optimization Algorithm
**GSR**	Galvanic skin response	Physiological Signal
**HDL**	Hybrid Deep Learning	-
**HHS**	Hilbert–Huang spectrum	Extracted Feature
**HMM**	Hidden Markov Model	Artificial Neural Network
**ICA**	Independent Component Analysis	Signal Processing tool
**iid**	independent identically distributed	Statistical Function
**LPF**	Low Pass Filter	Signal Processing tool (Filter)
**LSTM**	Long Short-Term Memory	Recurrent Neural Network Structure
**MESAE**	Multiple-fusion-layer based ensemble classifier of SAE	Deep Learning Neural Network
**ML**	Machine Learning	-
**MLP**	Multilayer Perceptron	Artificial Neural Network
**MTRBM**	Multichannel temporal Restricted Boltzmann Machine	Artificial Neural Network
**NN**	Neural Network	-
**OVR-FBCSP**	One-versus rest filter bank common spatial pattern	Signal Processing Tool
**P300**	Potential after 300 ms	Pattern in Electroencephalography
**PCA**	Principal Component Analysis	Signal Processing Tool
**PSD**	Power Spectral Density	Signal Measure
**RBN**	Restricted Boltzmann Machine	Artificial Neural Network
**ReLU**	Rectified Linear Unit	Activation function in Neural Networks
**RMSE**	Root Mean Square Error	Statistical Function
**RMSProp**	Root Mean Square Propagation	Optimization Algorithm
**RNN**	Recurrent Neural Network	Deep Learning Neural Networks
**RS**	Respiration signal	Physiological Signal
**SAE**	Stacked Autoencoder	Deep Learning Neural Networks
**SAM**	Selective Attention Mechanism	Feature Extraction tool
**SBD**	Stop Band Filter	Signal Processing tool (Filter)
**SGD**	Stochastic Gradient Descendent	Optimization Algorithm
**SI**	Speech Imagery	Task/Protocol
**SIMKAP**	Simultaneous capacity	Task/Protocol
**SNR**	Signal to Noise Ratio	Signal Measure
**SSAEP**	Steady-state Auditory Evoked Potential	Task/Protocol
**SSEP**	Steady-state Evoked Potential	Task/Protocol
**ST**	Skin temperature	Physiological Signal
**STFT**	Short-Time Fourier Transform	Signal Processing tool
**SVAE**	Stacked Variational AutoEncoder	Deep Learning Neural Networks
**VAE**	Variational Autoencoder	Deep Learning Neural Networks
**WAS-LSTM**	Weighted Average Spatial-LSTM	Deep Learning Neural Networks
**wICA**	wavelet-enhanced independent component analysis	Signal Processing tool

## Appendix B

Table A2 was designed to summarize relevant information from the reviewed articles such as database used, BCI application, techniques used to record the brain activity, task implemented, data preprocessing used, normalization used, features extracted, hDL architecture used, number of layers, optimization algorithm used and finally the performance obtained. All the different hDL architectures were classified into the following five subclasses: Deep Belief Network (DBN)-based, Convolution Neural Network (CNN)-based, Recurrent Neural Network (RNN)-based, CNN-RNN and CNN-DBN.

This table details the significant characteristics from the reviewed studies, as follow: (1) Dataset used in the studies, where local dataset indicates that the authors have recorded their own dataset, (2) The study application (N/A: study did not represent a clear application and focusing on the classification algorithm), (3) Techniques of bio-signal acquisitioning (BVP: Blood Volume Pressure, EEG: ElectroEncephaloGraphy, EMG: ElectroMyoGraphy, EOG: ElectroOculoGraphy, GSR: Galvanic Skin Response, RS: Respiration Signal, ST: Skin Temperature), (4) Task/Protocol of the experiment (MI: Motor Imagery, SIMKAP: simultaneous capacity, SSAEV: Steady State Auditory Evoked Potential, SSEV: Steady State Evoked Potential), (5) Pre-processing for cleaning and denoising the data (BPF: Band Pass Filter, CAR: Common Average Reference, CSP: Common Spatial Pattern, DAE: Denoising AutoEncoder, FIR: Finite Impulse Response, ICA: Independent Component Analysis, LPF: Low Pass Filter, PCA: Principal Component Analysis, SBF: Stop Band Filter), (6) Normalization (RMSE: Root Mean Square Error), (7) Extracting the features that represent the mental state (CCV: Channel Cross Variance, CNN: Convolutional Neuronal Network, DE: differential Entropy, DWT: Discreet Wavelet Transformation, HHS: Hilbert–Huang spectrum, PSD: Power Spectrum Density, SAM: Selective Attention Mechanism), (8) Architecture of the classification models: CNN-based: Convolutional Neuronal Network, CNN-DBN: Convolutional Neuronal Network-Deep Belief Network, CNN-RNN: Convolutional Neuronal Network-Recurrent Neuronal Network, DBN-based: Deep Belief Network, RNN-based: Recurrent Neuronal Network, (9) Number of layer of each architecture, (10) Optimization (ADAM: ADAptive Momentum, GWO: Gray Wolf Optimizer, RMSprop: Root Mean Square Propagation, SGD: Stochastic Gradient Descent), (11) Results: How the method are evaluated. For a better presentation, each dataset was put between parentheses.

**Table A2:** A survey of the selected studies in hybrid Deep Learning-based Brain-Computer Interface (hDL-based BCI).

Year	References	Database	Application	Training	Task	Pre-processing	Normalization	Feature extraction	Architecture	N° of Layers	Optimization	Results
**2015**	[23]	BCI competition IV	N/A	Offline	MI	Filter-Bank CSP (FBCSP) A bank of 9 filters from 4 to 40 Hz with a width of 4 Hz	N/A	Static & Dynamic Energy	CNN-Based	9	SGD	70.60%
**2015**	[12]	Local Dataset	Communication	Online	MI	N/A	Batch normalization	Selective Attention Mechanism (SAM)	RNN-Based	7	Adam Optimizer	93.63%
**2017**	[20]	Local Dataset BCI competition IV	Medical Care	Offline	MI	Filtering (BPF: Butterworth filter:0.5–50 Hz) DAE	N/A	Optical Flow from the EEG video	CNN-RNN CNN-RNN	8	N/A	72.22% 70.34%
**2017**	[43]	Local Dataset	Communication	Online	MI	Filtering (BPF: FIR: 1–200 Hz) CSP ICA	N/A	Variance	CNN-Based CNN-Based	27 27	SGD	70.80% 70.79%
**2017**	[25]	DEAP dataset	Emotion recognition	Offline	SSEP	Filtering (BPF: 4–45 Hz) ICA	Z-Score	425 silent physiological features from the 7 signals	DBN-Based	10	N/A	73.70%
**2017**	[46]	Local Dataset	N/A	Offline	P300	Filtering (BPF: FIR: 2–35 Hz) (SBF: 0.1 & 40 Hz)	N/A	Spatial and temporal features	CNN-RNN CNN-RNN CNN-RNN	10 15 15	Adam Optimizer	67.25% 68.75% 70.00%
**2017**	[70]	EEGmmidb	Communication	Online	MI	N/A	N/A	Spatial and temporal features	CNN-RNN	18	Adam Optimizer RMSPropOptimizer	95.53%
**2018**	[71]	Local Datasets BCI competition III BCI competition IV	N/A	Online	MI	Referencing Electrode Selection Artifact removal (ICA & PCA) Filtering (BPF: 8–12 Hz & 18–26 Hz)	Batch normalization	16 spatial features through CNN + DWT	CNN-RNN	8	N/A	87.36%
**2018**	[72]	EEGmmidb	N/A	Offline	MI	N/A	N/A	Spatial and temporal features	RNN-Based	14	Adam Optimizer	68.20%
**2018**	[58]	BCI competition IV	N/A	Offline	MI	Filtering (68 BPF: 4–40 Hz) CSP	Batch normalization	Variance (Abstracted Features through CNN)	CNN-Based	8	Adam Optimizer	81%.
**2018**	[65]	Local Dataset	Medical Care	Online	MI	Filtering (LPF: 40 Hz)	N/A	Abstracted Features through CNN	CNN-Based	13	Adam Optimizer	76.90%
**2018**	[59]	OpenMIIR	Medical Care	Online	SSAEP	Filtering 5 BPF (α: 8–13 Hz, β: 14–30 Hz, γ: 31–51 Hz, δ: 0.5–3 Hz, θ: 4–7 Hz)	N/A	Optical Flow from the EEG video	CNN-RNN	13	N/A	35%
**2018**	[73]	BCI competition II BCI Competition III	N/A	Offline	P300	Filtering (BPF: Butterworth filter: 0.1–30 Hz)	Z-Score	Spatial and temporal features	DBN-Based	4	Mini-batch	88.90%
**2018**	[69]	DEAP dataset	Emotion recognition	Offline	SSEP	Filtering BPF: Butterworth filter (α: 8–12 Hz, β: 12–30 Hz, γ: 30–100 Hz, θ: 4–8 Hz)	Z-score	Differential Entropy (DE)	CNN-RNN	6	Adam Optimizer	90.24%
**2018**	[74]	DEAP dataset	Emotion recognition	Offline	SSEP	N/A	Z-score	Spatial and temporal features	CNN-RNN	5	Adam Optimizer	91.03%
**2018**	[75]	DEAP dataset	Emotion recognition	Offline	SSEP	Filtering (BPF: 4–45 Hz)	N/A	(Statistical measures) (Power features) (Power differences) (Hilbert–Huang spectrum (HHS))	DBN-Based	7	N/A	76.36%
**2018**	[76]	Public (Bashivan, Bidelman, Yeasin EEG data set)	Mental state detection	Offline	Cognitive	Filtering (BPF 4–7, 8–13, 13–30 Hz)	N/A	High-level features	CNN-DBN	14 17	SGD	91.32% 92.37%
**2019**	[60]	BCI competition IV	N/A	Offline	MI	N/A	Batch normalization	Spatial and temporal features	CNN-RNN	9	Adam Optimizer SGD	59%
**2019**	[77]	BCI competition IV	N/A	Offline	MI	Filtering (16 BPF: Chebyshev Type II 4–38 Hz)	Truncated normal distribution function	Spatial and temporal features	CNN-RNN	8	Adam Optimizer	83%
**2019**	[66]	Local Dataset BCI competition IV	N/A	Offline	MI	Remove the average Filtering (BPF: 8–13 Hz)	N/A	Spatial Features	CNN-DBN	8	N/A	92%
**2019**	[78]	BCI competition IV	N/A	Offline	MI	Filtering (BPF: 0.5–100 Hz)	Batch normalization	Spatial and temporal features	CNN-RNN	18	Adam Optimizer	40%
**2019**	[79]	BCI competition IV	N/A	Offline	MI	Filtering (1 Hz-45 Hz based on Morlet wavelet transformation)	Batch normalization	Spatial and temporal features	CNN-Based	4	SGD	76.62%
**2019**	[41]	Local Dataset BCI competition IV	N/A	Offline	MI	Filtering (BPF: 6–13 & 17–30 Hz)	Batch normalization	Spatial Features through CNN	CNN-DBN	10	SGD	56.4 (Kappa)
**2019**	[80]	EEG based speech database	Medical Care	Offline	SI	N/A	N/A	Spatial and temporal features Channel cross-covariance (CCV)	RNN-Based	18	Adam Optimizer	79.98%
**2019**	[17]	EEGmmidb EEG-S TUH	Motor Imagery Recognition Person Identification (PI) Medical Care	Online	Cognitive	N/A	N/A	Spatial features	CNN-Based	5	Adam Optimizer	98.64%
**2019**	[57]	Local Dataset	Mental State Detection	Offline	Cognitive	N/A	N/A	DWT	CNN-Based	7	Adam Optimizer	92%
**2019**	[81]	(Exploiting P300 Amplitude changes) (BCI Competition III) (Auditory multi-class BCI) (BCI-Spelling using Rapid Serial Visual Presentation) (Examining EEG-Alcoholism Correlation) (Decoding auditory attention)	N/A	Offline	P300	Filtering (BPF: 0.15–5 Hz 0.1–60 Hz 0.1–250 Hz 0.016–250 Hz 0.02–50 Hz 0.016–250 Hz)	Batch normalization	Spatial and temporal features	DBN-Based	62	RMSprop optimizer	79.37% 88.52%
**2019**	[26]	Local Dataset	Mental stateDetection	Offline	Cognitive	Filtering (BPF: Butterworth filter 1–50 Hz) ICA	Batch normalization	Spatial and temporal features	CNN-RNN	23	N/A	87%
**2019**	[11]	Local dataset Public dataset	Communication	Offline	SI	N/A	N/A	Spatial and temporal features	CNN-RNN	6	N/A	95.53%
**2019**	[82]	Local	Person identification	Offline	Resting state	DWT	Batch Normalization	Temporal features	RNN-Based	9	N/A	95.60%
**2019**	[83]	Local	Comunications (Robotics)	Online	MI	Filtering (LPF 40 Hz)	Batch Normalization	Spatial features	CNN-Based	19	Adam Optimizer	76.90%
**2019**	[84]	Public (Bashivan, Bidelman, Yeasin EEG data set)	Mental state detection	Offline	Cognitive	Filtering (BPF 0–7, 7–14, 14–49 Hz)	N/A	Spatial temporal frequency features	CNN-RNN	13	RMSProp Optimizer	96.30%
**2020**	[85]	Local Dataset	Medical Care	Online	MI	Filtering (BPF: 0.2 Notch filter: 60 Hz)–45 Hz	Mini-max normalization	Temporal features	RNN-Based	6	Adam Optimizer	97.50%
**2020**	[86]	MAKAUT Dataset AI Dataset	Emotion recognition	Online	SSEP	Filtering (BPF 10 order: Chebyshev)	N/A	(Time domain EEG features) (Frequency domain EEG features) (Time-frequency domain EEG features) (The standard CSP features)	RNN-Based	6	Adam Optimizer	88.71%
**2020**	[10]	EEGmmidb	N/A	Offline	MI	N/A	Batch normalization	Spatial and temporal features	RNN-Based	13	Adam Optimizer	98.81% 94.64%
**2020**	[42]	DEAP dataset	Emotion recognition	Online	SSEP	Filtering (BPF: 4–47 Hz) Common average referencing ocular artifacts removing by blind source separation algorithms	Z-score	Spatial and temporal features PSD	CNN-RNN	7	Adam Optimizer	93.20% 93.00%
**2020**	[64]	(Graz University Dataset) (BCI competition IV)	N/A	Offline	MI	Filtering (BPF: 8–24 Hz, 8–30 Hz, 8–40 Hz)	Batch normalization	Spatial and temporal features	CNN-Based	19	Adam Optimizer	76.07%
**2020**	[4]	BCI competition IV	N/A	Offline	MI	Filtering notch filter 50 Hz)	Batch normalization	Temporal features	CNN-RNN	8	Adam Optimizer	95.62%
**2020**	[87]	BCI competition IV	N/A	Offline	MI	Filtering (FBCSP: 12BPF: 6–40 Hz) Hilbert transform algorithm	Batch normalization	Spatial features	DBN-Based	29	N/A	0.630 Kappa
**2020**	[88]	DEAP dataset	Emotion recognition	Offline	SSEP	Filtering (BPF: 4–45 Hz)	Batch normalization	Spatial and temporal features	CNN-RNN	9	Adam Optimizer	99.10% 99.70%
**2020**	[89]	BCI competition IV	N/A	Offline	MI	Filtering (BPF 4th order Butterworth 4–7 Hz, 8–13 Hz, 13–32 Hz)	N/A	High-level features	CNN-Based	5	N/A	74.60%
**2020**	[90]	BCI competition III	Communication	Online	MI	Filtering (BPF: FIR: Hamming-windowed: 4–40 Hz) ICA Common average reference (CAR)	RMSE (root mean square error)	Spatial and temporal features	CNN-RNN	9	Adam Optimizer	0.6 0.43 Success rates
**2020**	[91]	EEGmmidb	N/A	Offline	MI	Filtering (BPF: 8–13 Hz &13–30 Hz)	N/A	Spatial and temporal features	CNN-RNN	20	SGD	82.10% 83.50%
**2020**	[92]	BCI competition IV	Data Augmentation	Offline	MI	Filtering (BPF: 8–30 Hz) Spectrogram	Batch normalization	Images features from Spectrogram	CNN-Based	24	Adam Optimizer	126.4 98.2 (FIDs)
**2020**	[93]	BCI competition IV	Person identification	offline	MI	Filtering (Chebyshev 4–8 Hz, 8–12 Hz...)	Truncated normal distribution	Spatial and temporal features	CNN-RNN	13	Adam Optimizer	Kappa 0.8
**2020**	[94]	“STEW” dataset	Mental state detection	Offline	“No task” & (SIMKAP)	Filtering (BPF 4–32 Hz)	Batch Normalization	(Frequency features (PSD)) (Linear domain features (Autoregressive coefficient)) (Non -Linear domain features (approximate entropy, Hurst Exponent) (Time domain)	RNN-Based	11	Gray Wolf Optimizer (GWO)	84.45%
**2020**	[95]	BCI competition IV Local Dataset	N/A	Offline	MI SI	Filtering (Butterworth BPF 4–35 Hz)	Batch Normalization	Temporal-spatial-frequency features	CNN-RNN CNN-RNN CNN-RNN CNN-RNN	20 15 20 15	Adam Optimizer	86% 82% 82% 71% Kappa: 0.64

## Appendix C

### Appendix C.1. Deep Learning Overview

The starting point to DL algorithms is Multilayer Perceptron (MLP) which is an Artificial Neural Network (ANN) with more than one hidden layer, and this represents the simplest DL algorithm. DL algorithms are categorized into discriminative, representative, generative and hybrid.

Discriminative algorithms are used for combining feature extraction and classification steps and act as supervised learning including Convolutional Neural Network (CNN) and Recurrent Neural Network (RNN). Firstly, CNN is used to extract the hidden latent spatial information, from images mainly, and classify that information depending on the fully connected layer and SoftMax layer (a decision-making layer). The main structure of CNN is composed of a series of convolutional and pooling layers with different parameters. Secondly, RNN is a neural network that receives information not only from the present state but also from the previous state. This unique feature of RNN makes it excellent for time-related problems. Two substructures of RNN are used, Long Short-Term Memory (LSTM) and Gated Recurrent Units (GRU).

Representative algorithms are used for encoding features from the input and work as unsupervised learning. The simplest algorithm is Autoencoder (AE) which is consists of symmetric input and output layers and hidden layer, more than two hidden layers to be considered as Deep Autoencoder (D-AE) and it must be an odd number of layers. By making the weights of the connection input-hidden and hidden-output similar, Restrict Boltzmann Machine (RBM) is defined. It is represented as one visual layer, input, and output together, and hidden layers with bidirectional connection all along with the network. By Stacking AEs or RBMs, we got a new kind of network known as Deep Belief Network (DBN).

Generative algorithms are not yet widely applied in BCIs. It could be considered as an enhanced model of AEs with probabilistic features. Hybrid deep learning algorithms are obtained by combining two or more of the simple deep learning algorithms.

### Appendix C.2. Deep Belief Networks-Based Hybrid Deep Learning Algorithms

Previous Hybrid DBN-based methods used in BCI systems could be categorized into three structures:

- DBN assisted by Glia cells (GC-DBN)
- Multiple-fusion-layer based ensemble classifier of stacked autoencoders (MESAE)
- Event-Related Potential Network (ERP-NET)

#### Appendix C.2.1. DBN Assisted by Glia Cells (GC-DBN)

This method improves the DBN by adding assisting Glia Cells (GC). Since DBN is a group of stacked RBMs, each glia cell is connected to a unit in the hidden layer of RBM. GC could be considered as a thresholding reference for activating each neuron. The GC activity depends on the corresponding neuron, i.e., the GC passes or decays the activation, depending on whether the corresponding neuron signal reaches a prespecified threshold, before conveying it to the next GC.

From a mathematical point of view, the activation functions turn into Equation (A1)

h_j_ = σ (h_j_* ^+^α.g_i_), (A1)

where h_j_ is the output of the hidden node j. σ is the activation function, h_j_* is related to the connection weight of the visual unite i and the hidden unit j, α is the weight coefficient of glia effect value, g_i_ is the glia effect value.

Despite the fact that DBN is hard to work for mining inter-frequency and inter-channel correlation information, the role of GCs in DBN can overcome this issue in emotion recognition BCI systems [75].

#### Appendix C.2.2. Multiple-Fusion-Layer Based Ensemble Classifier of Stacked Autoencoders (MESAE)

This method depends on using 3-layers stacked autoencoders (SAEs), and its output is fed to a feature fusion network creating multiple-fusion-layer based ensemble classifier of SAEs (MESAE).

To create this network, three steps are required:

- Initialize member SAEs.
- Model structure identification for member SAEs.
- Construct a hierarchical feature fusion network.

This hybrid deep learning method was applied for emotion recognition task.

It has a higher generalization capability than the shallow emotion recognition methods due to its complexity in comparison with the shallow ones.

One previous experiment on this net depended on six sets of abstracted features extracted from EEG, Electrooculography (EOG), Electromyography (EMG), skin temperature, galvanic skin response (GSR), blood volume pressure and respiration signal by using a specific way related to K-means clustering [25,81].

#### Appendix C.2.3. Event-Related Potential Network (ERP-NET)

This method aims to detect the ERP patterns in EEG signals depending on the temporal and spatial pattern. The method name is ERP-Net which consist of five layers of Multichannel Temporal Restricted Boltzmann Machine (MTRBM). The network was tested on data set IIb in BCI competition II and data set II in BCI Competition III and the results were compared against many other algorithms, namely, SVM, CNN, Lasso, BLDA, STDA, gLasso and gsBLDA. The ERP-NET could be considered as a promising analytical tool for the research on ERP signals [73].

### Appendix C.3. CNN-Based Hybrid Deep Learning Algorithms

Convolutional Neural Networks is one of the most admired deep learning models specialized in spatial information exploration. CNN is widely used, in the reviewed literature, to discover the latent spatial information in applications such as the analysis of motor imagery data [40], robotics [65,83]. increasing the learning capacity of BCI systems [58], detecting depression with EEG signals and to evaluate a novel deep learning method for classifying binary motor imagery data [41].

Some studies propose new network structures that mix CNN with representation algorithms for feature extraction and classification. Firstly, linear and nonlinear classifiers merge the simplicity of machine learning algorithms and the efficiency of CNN since it avoids the traditional feature engineering process by learning high-level features automatically. Linear classifiers collect discriminant classifiers that use linear decision boundaries between the feature vectors of each class. They include Linear Discriminant Analysis (LDA), and Support Vector Machines (SVMs) [23]. On the other hand, Nonlinear Bayesian classifiers are classifiers modelling the probability distributions of each class and use the Bayes rule to select the class to assign to the current feature vector. The Hidden Markov Model (HMMs) [58] can be represented as the simplest dynamic Bayesian network. Secondly, Neural Networks (NN) [43] can be used to approximate any non-linear decision boundary. A type of NN is the Multi-Layer Perceptron (MLP), typically employing only one or two hidden layers and long-short term memory (LSTM). Finally, probabilistic-based methods are used to capture the most hidden features of the training data such as stacked autoencoder (SAE) [40] and Variational autoencoder (VAE) [41]. As a whole overview of hybrid-DL Based BCIs, according to the type of combination, boosting, voting, or stacking, we can note that the combination of these methods results in consistent increases of the accuracy in almost all studies. Lastly, the classifier combination seemed to be the best performing classifiers for EEG-based BCIs.

A further method depended on Reinforced-CNN aims to classify Cognitive Activity Recognition into Movement Intention Recognition (MIR), Person Identification (PI) and Neurological Diagnosis (ND), this method goes into extracting the robust and distinct deep features automatically by combining the deep reinforcement learning and attention mechanism. The proposed Reinforced CNN selects the best attention area that leads to the highest classification accuracy using a non-linear reward function to encourage the model. Comparing the results of this method with literature shows an improvement of 2.3% in classification accuracy an average over three datasets [96].

Another companion to CNN is a Genetic algorithm. This method based on Merging CNN with an Evolutionary Algorithm (EA) to classify EEG signals when seeing an object (Visible Mode) and imagining an object (Invisible Mode). The proposed models filter the output of CNN using Discrete Wavelet Transform (DWT) with Coiflet wavelet mother signal. And the output of the filter is fed into an Age-Layered Population Structure (ALPS) Genetic Algorithm (GA). The results compared with five CNN-based algorithms resulting in 92% accuracy [57].

### Appendix C.4. RNN-Based Hybrid Deep Learning Algorithms

The use of Hybrid-DL based on RNN has two main structures:

- Weighted Average Spatial-LSTM (WAS-LSTM)
- Stacked RNN

#### Appendix C.4.1. Weighted Average Spatial-LSTM (WAS-LSTM)

It focuses on the spatial dependency between different dimensions at the same time-point instead of the temporal dependency between a sequence of samples collected at different time-points in normal LSTM [12].

To obtain the optimal dependency which includes the most distinctive activates was proposed alternative composed of three components:

- The autoregressive model.
- The Silhouette Score.
- The reward functions.

The main reasons for WAS-LSTM usage:

1. To capture the cross-relationship among feature dimensions, which is extracted using Selective Attention Mechanism (SAM), in the optimized focal zone.
2. It could stabilize the performance of LSTM via average methods.

This makes WAS-LSTM an efficient method.

#### Appendix C.4.2. Stacked RNN

RNN is extended by LSTM by adding three gates to an RNN, which enable LSTM to learn long-term dependency in a sequence and make it easier to be optimized [72,85].

A bidirectional LSTM is a combination of two normal LSTM which allows dependencies in the reverse directions, so it encodes spatial information, in comparison with standard LSTM that flows in the forward time direction and encodes temporal information.

Consequently, the combination of the two types encodes both temporal and spatial information.

The steps following are applied in order to meet two conditions, independent identically distributed (i.i.d) and to fully utilize the RNNs’ potential:

1. Rearrange the index of recorded electrodes according to their spatial positions so the data can be viewed as a spatial sequential stream.
2. Spilt the samples according to the trial index.
3. A parallel RNNs model was proposed.

The advantages of this method could be summarized by transforming EEG into a spatial and temporal sequence which outperforming older methods by over 8.25% in intention recognition accuracy.

### Appendix C.5. CNN-RNN Hybrid Deep Leering Algorithms

A great method to extract spatial and temporal features from raw EEG signal is the combination of two mostly used DL algorithms, CNN and RNN. CNN-RNN structure is used in many BCI systems, which could be divided into different specialized structures as follows.

The first one is the IncepCNN built combining BGRU as RNN structure with CNN. Four levels of the convolutional kernel are stacked, with ReLU activation function, followed by a fully connected layer. Afterwards, it is used BGRU, a bidirectional mechanism to transfer information in both directions. Thanks to this hybrid CNN-BGRU all features extracted can be well separated [26,79].

On the same side, there is the CNN-LSTM-DWT-BN structure that fuses spatial-temporal features to catch temporal correlation and incorporate it into the system. It combines a hierarchical feature extractor and spatial convolution layer with DWT and LSTM that capture all temporal dynamics in the data [69,71] Very similar to this, is CRAM, a Convolutional Recurrent Attention Model that uses CNN to encode the representation of EEG signals, starting with Encoded EEG Temporal slices, a mechanism is introduced to discover the attentive temporal dynamics of them, using also LSTM to construct RNN layers [60].

Another way is followed by the hybrid structure that converts EEG signal into an EEG Video following many steps:

- projecting the 3D locations of the electrodes into two dimensions using azimuthal equidistant projection (AEP);
- interpolating these locations into a 2D grey image;
- Show those images in a timeline that produce the EEG-video.

This technique allows to extract information from the video and applied to a symmetric CNN. Afterwards, the output of RNN passes through two RNNs (LSTM or GRU) with a memory cell after being reshaped. [20,78].

Up to date, BCI system depends on Speech Imagery to be decoded using EEG signal depending on channel cross-covariance (CCV). The algorithm called Hierarchical Structure is based on three hierarchical levels. Firstly, the first four layers are two different independent networks. One branch is CNN, and the other branch consists of two fully connected hidden layers stacked with two LSTM layers. Secondly, the fifth layer is the deep autoencoder layer followed by the fully connected layer. Finally, the output layer is represented by a SoftMax layer [80]. It is also important to reduce the time-training of a Deep classification, to do that there is a Deep Transfer Learning framework built on two steps to achieve the final EEG category labels. Starting from EEG data, all features are extracted by the transfer network. After that, the features pass in two RNN layers, two full-connected layers and LSTM to avoid vanishing gradient problems during the training. In the end, are applied two full-connected layers and the last layer use a SoftMax to achieve the final EEG label [11,59].

For classifying P300 BCI Signals, there are also hybrid structures like the Multidimensional CNN that combine a deep hierarchical feature extractor with the one that can learn to recognize and synthesize the temporal features. This structure depends on combining different architectures of 2D-CNN and 3D-CNN with RNN-LSTM [46,69].

The hybrid structures could be used for many aims, one of these is to decodes the brain activity into a text with the ability to implicate this method in real-world applications. Depends on a framework that merges CNN with RNN using a dataset called Eegmmidb, this method results in an accuracy of 95.53% [70]
